# Deep learning empowering design for selective solar absorber

**DOI:** 10.1515/nanoph-2023-0291

**Published:** 2023-08-11

**Authors:** Wenzhuang Ma, Wei Chen, Degui Li, Yue Liu, Juhang Yin, Chunzhi Tu, Yunlong Xia, Gefei Shen, Peiheng Zhou, Longjiang Deng, Li Zhang

**Affiliations:** National Engineering Research Center of Electromagnetic Radiation Control Materials, Key Laboratory of Multi-spectral Absorbing Materials and Structures of Ministry of Education, University of Electronic Science and Technology of China, Chengdu, 611731, China; Institute of Electromagnetics and Acoustics and Key Laboratory of Electromagnetic Wave Science and Detection Technology Xiamen University Xiamen, Fujian 361005, China; School of Ocean Information Engineering, Jimei University, Xiamen 361021, China

**Keywords:** deep-learning, metasurface, solar absorber

## Abstract

The selective broadband absorption of solar radiation plays a crucial role in applying solar energy. However, despite being a decade-old technology, the rapid and precise designs of selective absorbers spanning from the solar spectrum to the infrared region remain a significant challenge. This work develops a high-performance design paradigm that combines deep learning and multi-objective double annealing algorithms to optimize multilayer nanostructures for maximizing solar spectral absorption and minimum infrared radiation. Based on deep learning design, we experimentally fabricate the designed absorber and demonstrate its photothermal effect under sunlight. The absorber exhibits exceptional absorption in the solar spectrum (calculated/measured = 0.98/0.94) and low average emissivity in the infrared region (calculated/measured = 0.08/0.19). This absorber has the potential to result in annual energy savings of up to 1743 kW h/m^2^ in areas with abundant solar radiation resources. Our study opens a powerful design method to study solar-thermal energy harvesting and manipulation, which will facilitate for their broad applications in other engineering applications.

## 1 Introduction

The overexploitation of fossil fuels and global warming are significant challenges in our society today. As one of the most promising renewable resources, solar energy has garnered tremendous interest for its potential to address these issues. Among the various studies in this field, solar metamaterial absorbers have shown great promise for converting sunlight into available energy, with potential applications in thermophotovoltaics [1–7], seawater desalination [8–10], and sewage purification [11, 12]. To maximize the utilization of solar energy, it is crucial to establish an ideal solar absorber. According to Wien’s displacement law [13], an ideal solar absorber should have high absorption in the solar spectrum (0.3–2.5 µm) and zero emissivity in the infrared region (2.5–25 µm), preventing heat leakage and optimizing its performance as a photothermal energy conversion device. In essence, solar absorbers must exhibit exceptional spectrum selectivity. In recent years, numerous studies have emerged on the topic of selective solar absorber design. Various structure systems have been proposed for selective solar absorbers, including densely packed nanowires [14], nanotubes [15], nanopillars [16], 1D or 2D gratings [17], sawtooth and pyramid structures [18–23], and different periodic patterns [24–34]. However, these designs primarily rely on brute-force, time-consuming, and inefficient trial-and-error conventional domain-knowledge-driven design strategies, resulting in a suboptimal performance for selective solar absorbers.

Layered film photonic structures offer several advantages for the development of selective solar absorbers, including their simple planar geometry, precisely controllable dimensions, flexibility in combining different materials, and potential for large-scale manufacturing. Several optimization schemes, such as genetic algorithms (GA) [35, 36] and particle swarm algorithms (PSA) [37–39], have been developed and applied to design layered film photonic structures for optical and selective solar absorbers. Up to date, these current optimization methods suffer from several problems: (i) Intelligent optimization algorithms rely on electromagnetic simulation calculations for data acquisition, and higher optimization accuracy requires larger simulation calculations, significantly increasing the optimization process’s time cost; (ii) Popular intelligent optimization algorithms like particle swarm and genetic are prone to settling on local optimal solutions when faced with high-dimensional optimization problems; (iii) Current optimization methods primarily focus on achieving single-objective optimization and are incapable of solving multi-objective optimization problems, such as those found in spectrally selective absorbers. Thus, it remains in-demand to pursue a powerful scheme to accelerate the development of ideal selective solar absorbers.

Here, we report the development of an ideal selective solar absorber (SSA) using a deep-learning (DL) architecture aided by multi-objective double annealing (DA) algorithm optimization scheme. Utilizing the DL architecture, the relationship between complex nanostructure parameters and optical response spectra can be real-time predicted, eliminating iterative and time-consuming calculations. When the DA algorithm is used for the global optimization, the DL network can predict the optical responses in the time scale of millisecond, significantly speeding up design process. The algorithm also offers various candidates, enabling the selection of suitable material and thickness of each layer to meet desired optical experiment requirements. We fabricate a wafer-scale SSA with broadband sunlight absorption via DL architecture aided by multi-objective DA algorithm optimization scheme. The on-site experiments under the sunlight indicate that our SSA is promising for photothermal conversion. Our study provides a powerful design way to develop SSA and various other metamaterial-based applications.

## 2 Results and discussion

**Figure 1:**
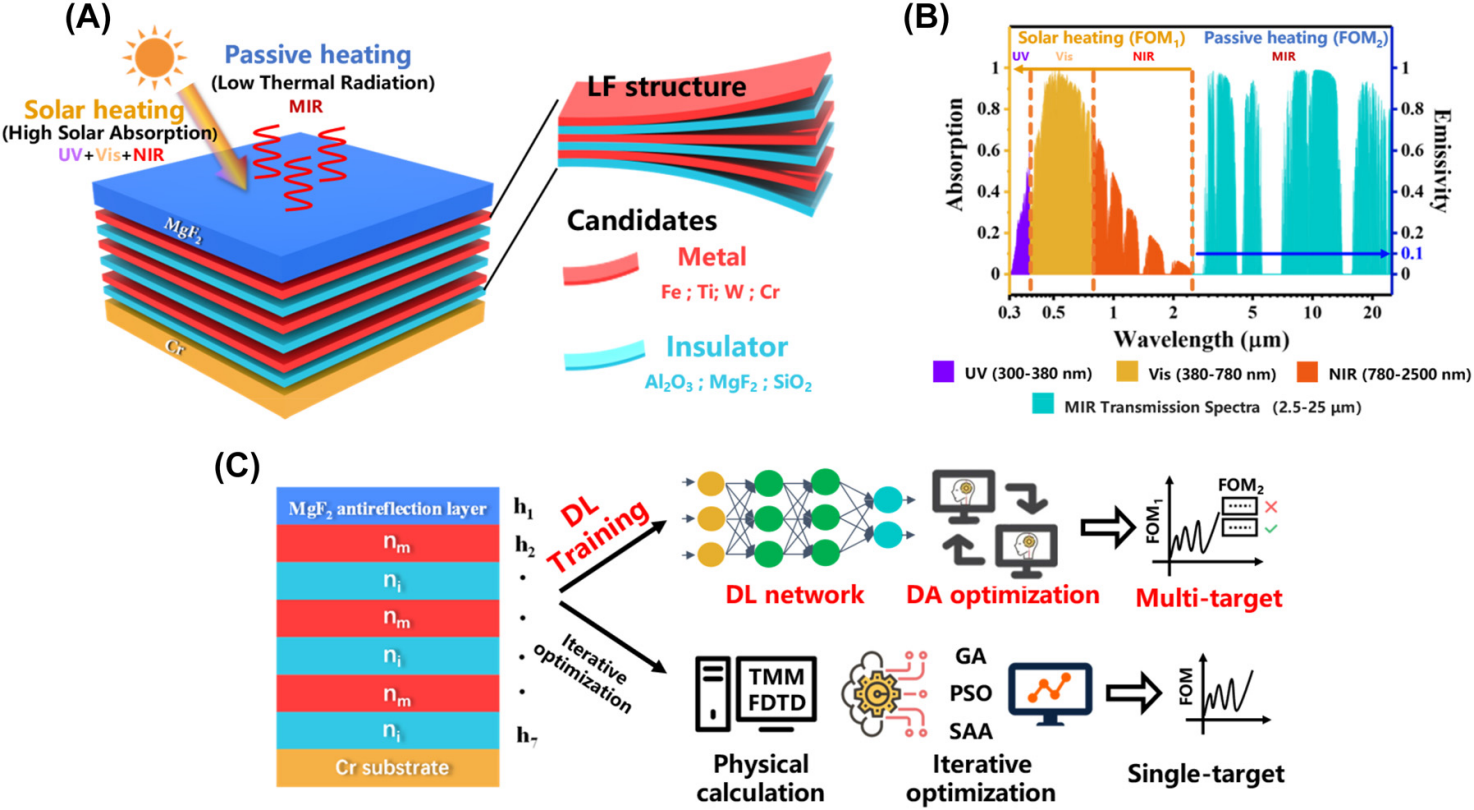
SSA concept and the DL-aided multi-objective optimization scheme. (A) The schematic structure of the LF in the SSA containing candidate materials. (B) The solar spectrum (air mass 1.5 global), the resultant transmitted irradiance (blue shade), and the absorption/emissivity of an SSA in relevant wavelength regimes. The SSA should have high absorption in the UV to NIR (300−2500 nm) spectrum for solar heating. Additionally, it should have low emissivity in the MIR (2.5–25 μm) for passive heating. (C) Schematic comparison of DL-aided multi-objective DA optimization scheme and current conventional optimization scheme.

The candidate SSA consists of layered film (LF) photonic structures on a chromium (Cr) substrate with a magnesium fluoride (MgF_2_) anti-reflection layer (Figure 1A). The low-refractive-index MgF_2_ layer on top acts as an anti-reflection layer, reducing the reflection of the incident light from the absorber and increasing the amount of light that can enter the absorber, while the Cr substrate acts as an opaque reflector. The LF structure is made of four common metal materials and three common insulator materials, where the ultra-thin metallic layer acts as a translucent mirror together with the lossless insulator layer and the Cr substrate at the bottom to form a metal–insulator–metal–insulator–metal (MIMIM) structure, so their combination has the potential to produce different optical properties (high absorption and low emissivity) in different wavelength regimes. As shown in Figure 1A, the LF structure is divided into 6 layers, metallic layers can be one of the four candidate materials: iron (Fe), titanium (Ti), tungsten (W), and Cr, and insulator layers can be one of the three candidate materials: aluminum oxide (Al_2_O_3_), MgF_2_, and silicon dioxide (SiO_2_). All metal and insulator layers are set as fixed materials, so there are a total of twelve material combinations. It is designed to absorb the majority of energy from solar radiation while preventing heat leakage through the mid-infrared range (MIR) (Figure 1B). This allows for the efficient coupling of solar and passive heating to maximize the heating effect. Under the classical design framework, such complex combinations require brute-force optimizations with sizable parameter space, which turn out to be rather inefficient and time-consuming.

With the purpose of overcoming this challenge, we develop a DL-aided multi-objective DA algorithm optimization scheme. The DA algorithm is an extended algorithm based on the traditional simulated annealing algorithm [40]. It uses the annealing procedure from the traditional annealing algorithm and combines the local search algorithm with the global search algorithm. Global search algorithms typically perform well at identifying basins (areas in the search space) where the best solution may be found, but they frequently struggle to find the best solution within the basin. On the other hand, local search algorithms excel at identifying the basin’s ideal value. The DA algorithm can run multiple annealing at the global scale, choose the best result from them, and then recursively run the search again by focusing on that result until the annealing no longer yields an optimal result or the new solution is hardly better superior. In the multi-objective optimization process, the weighted absorption of the solar spectrum (figure-of-merit-1, FOM_1_) as the main optimization objective and the average MIR emissivity (FOM_2_) as the constraint. In terms of practical applications, an SSA should possess both high solar spectral absorptance and low thermal emissivity in the infrared region, in accordance with Planck’s law of blackbody radiation. The spectral density of thermal radiation from a blackbody absorber can be defined by [41]:

(1) $$B_{\lambda }\left(\lambda ,T\right)=\frac{2hc^{2}}{\lambda^{5}}\frac{1}{e^{\frac{hc}{\lambda kT}}-1}$$

where λ is the wavelength, *T* is the absolute temperature of the blackbody absorber, his Planck’s constant, *c* is the speed of light in the vacuum, and *k* is the Boltzmann constant. According to the formula, we calculate the irradiance spectra at different temperatures in Figure S1 (Supporting Information). Thus, the FOM_1_ and FOM_2_ can be defined as:

(2) $${\text{FOM}}_{1}=\frac{\int_{0.3}^{2.5}I_{\text{AM}1.5}\left(\lambda \right)\alpha \left(\lambda \right)\text{d}\lambda }{\int_{0.3}^{2.5}I_{\text{AM}1.5}\left(\lambda \right)\text{d}\lambda }$$

(3) $${\text{FOM}}_{2}=\frac{\int_{2.5}^{25}\varepsilon \left(\lambda \right)\text{d}\lambda }{25-2.5}$$

where the $I_{\text{AM}1.5}\left(\lambda \right)$ [42] denotes the direct normal irradiance of solar light at the wavelength *λ*, $\alpha \left(\lambda \right)$ and $\varepsilon \left(\lambda \right)$ denote the direct normal spectral absorption and emissivity of the SSA, respectively. The multi-objective optimization problem is formulated as follows:

(4) $$\left\{\begin{array}{c} {\text{Maximize FOM}}_{1}\\ {\text{Subject to FOM}}_{2}≦0.1 \end{array}\right.$$

Maximizing FOM_1_ while satisfying the FOM_2_ constraint is crucial to enable the designed SSA to approach the ideal SSA. The detailed workflow of the multi-objective DA algorithm optimization scheme is illustrated in Figure S2 (Supporting Information). The relationship between the SSA and its FOM is described by a DL network that is trained using a dataset generated by FDTD. The dataset generation process is outlined in Table S1 and Figure S3 (Supporting Information).

The use of DL has brought significant benefits, as demonstrated in Figure 1C. Compared to conventional optimization methods, the DL-aided multi-objective DA optimization scheme drastically reduces the time required for complex simulation calculations, making optimization several orders of magnitude faster, especially when the SSA complexity is high or when multiple optimization objectives are involved.

Here, we utilize DL-aided multi-objective optimization scheme to perform the inverse design of an SSA. Our goal is to achieve maximum absorption in the solar spectrum while minimizing emissivity in the infrared region (Equation (4)).

**Figure 2:**
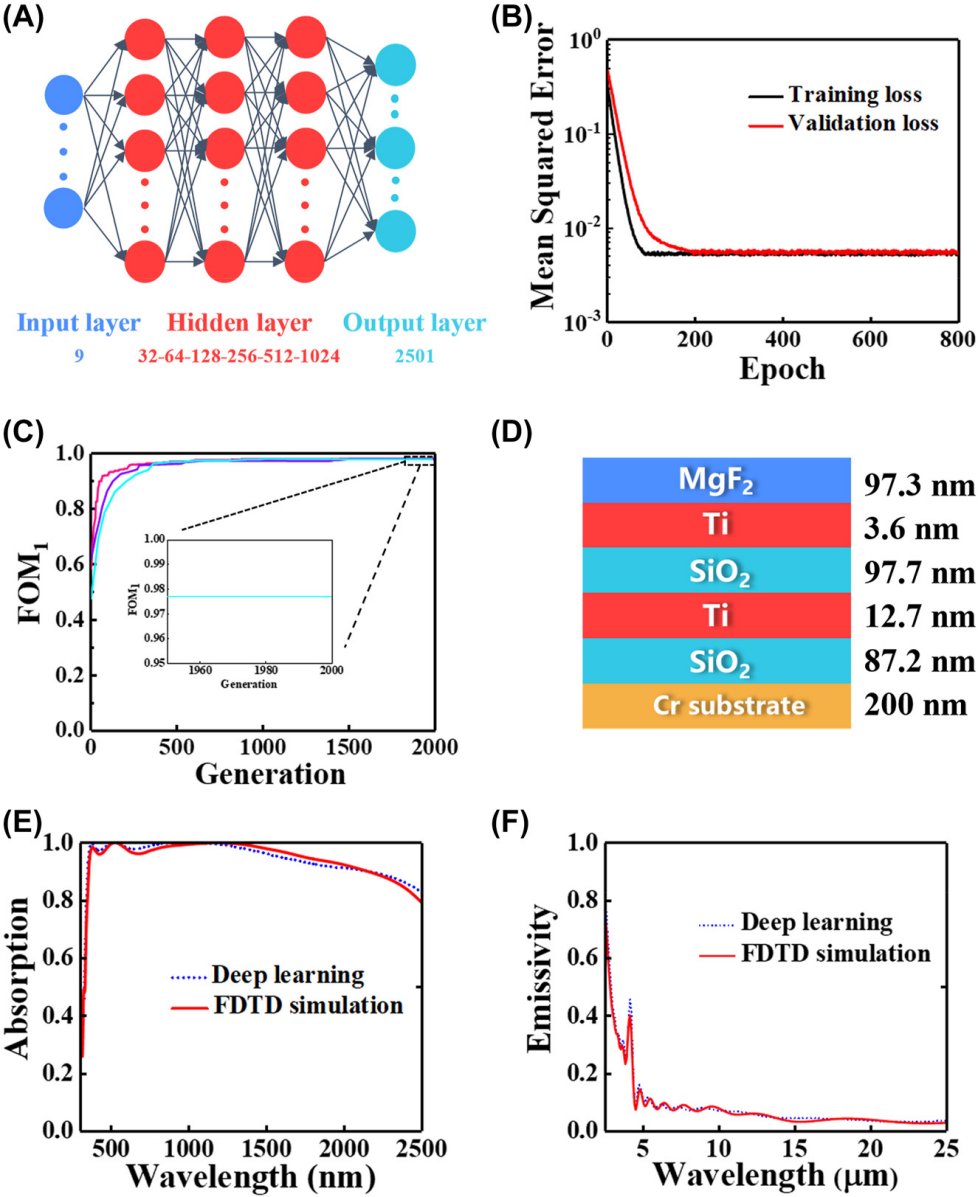
Optimization of the design process for SSA. (A) The architecture of the DL network. The DL network is composed of an input layer that corresponds to 9 design parameters, 6 hidden layers, and an output layer that corresponds to the 2501 spectral points. The number of neurons in the hidden layers is shown; (B) MSE of training and validation. After 800 epochs, the training and validation losses stabilize at 0.00556 and 0.0054, respectively; (C) optimization of historical FOM_1_ records of the SSA with three different randomly selected initial values; (D) optimal configuration for the SSA; (E) and (F) represent the absorption/emissivity calculated by the DL network (blue line) and FDTD (red line) in the solar spectrum and infrared region, respectively.

The most popular DL framework, TensorFlow, was used to build and train the DL Network. 9 parameters are selected as the input parameters vector *P* = [*h*_1_, *h*_2_, *h*_3_, *h*_4_, *h*_5_, *h*_6_, *h*_7_, *n*_*m*_, *n*_*i*_], including 7-dimensional unit-cell parameters and 2-dimensional material types of those parameters (Figure 2A). Before training, the dataset is randomly divided, with 80 % being used as the training set and 20 % being used as the validation set and the K-fold cross-validation is used for training. During the DL network training, six fully connected layers were used for deep learning training with 32, 64, 128, 256, 512, and 1024 neurons in each hidden layer. Random regularization is added after each layer of the network to deactivate some random neurons in the DL network, which improves the overfitting phenomenon, and the magnitude of regularization increases with the number of network layers. We choose the swish function as the activation function of the hidden layer. In order to obtain a faster convergence speed, we choose the Nadam òptimizer as the backpropagation optimizer. The mean squared error (MSE) between the predictive and actual absorptance is expressed to present the loss function:

(5) $${\text{Loss}}_{\text{MSE}}=\frac{1}{N}\overset{N}{\underset{i=1}{\sum }}{\left(\alpha_{\text{ANN}}\left(\lambda_{\text{i}}\right)-\alpha_{\text{FDTD}}\left(\lambda_{\text{i}}\right)\right)}^{2}$$

where $\alpha_{\text{ANN}}\left(\lambda_{i}\right)$ is the spectrum predicted by the DL network, $\alpha_{\text{FDTD}}\left(\lambda_{i}\right)$ is the simulated spectrum and *N* is the number of datasets. As shown in Figure 2B, both the training loss and the validation loss decay rapidly to a stable value at 200 epochs, after which the iterations are allowed to continue up to 800 epochs to reduce the value of MSE. The training and validation losses after 800 epochs are 0.00556 and 0.0054, respectively.

The DA algorithm is utilized for 2000 iterative calculations to obtain the optimal configuration, and the FOM_1_ of the optimization process is depicted in Figure 2C. Upon optimization to 2000 generations, FOM_1_ remains at 0.977 and the optimal parameter set is shown in Figure 2B. We have also optimized the LF structure using three different DA algorithms with randomly selected initial values, and in all cases, they converged to the same optimal structure (Figure 2D), although the number of iterations required was different in each case.

The absorption/emissivity calculated by FDTD and DL network for the final optimized structure is shown in Figure 2E and F. The FDTD and DL network results of FOM_1_ and FOM_2_ are 0.981/0.977 and 0.087/0.082, respectively. It can be seen that both in the solar spectrum and infrared region, the results calculated by FDTD and generated by the DL network maintain a high degree of agreement.

**Figure 3:**
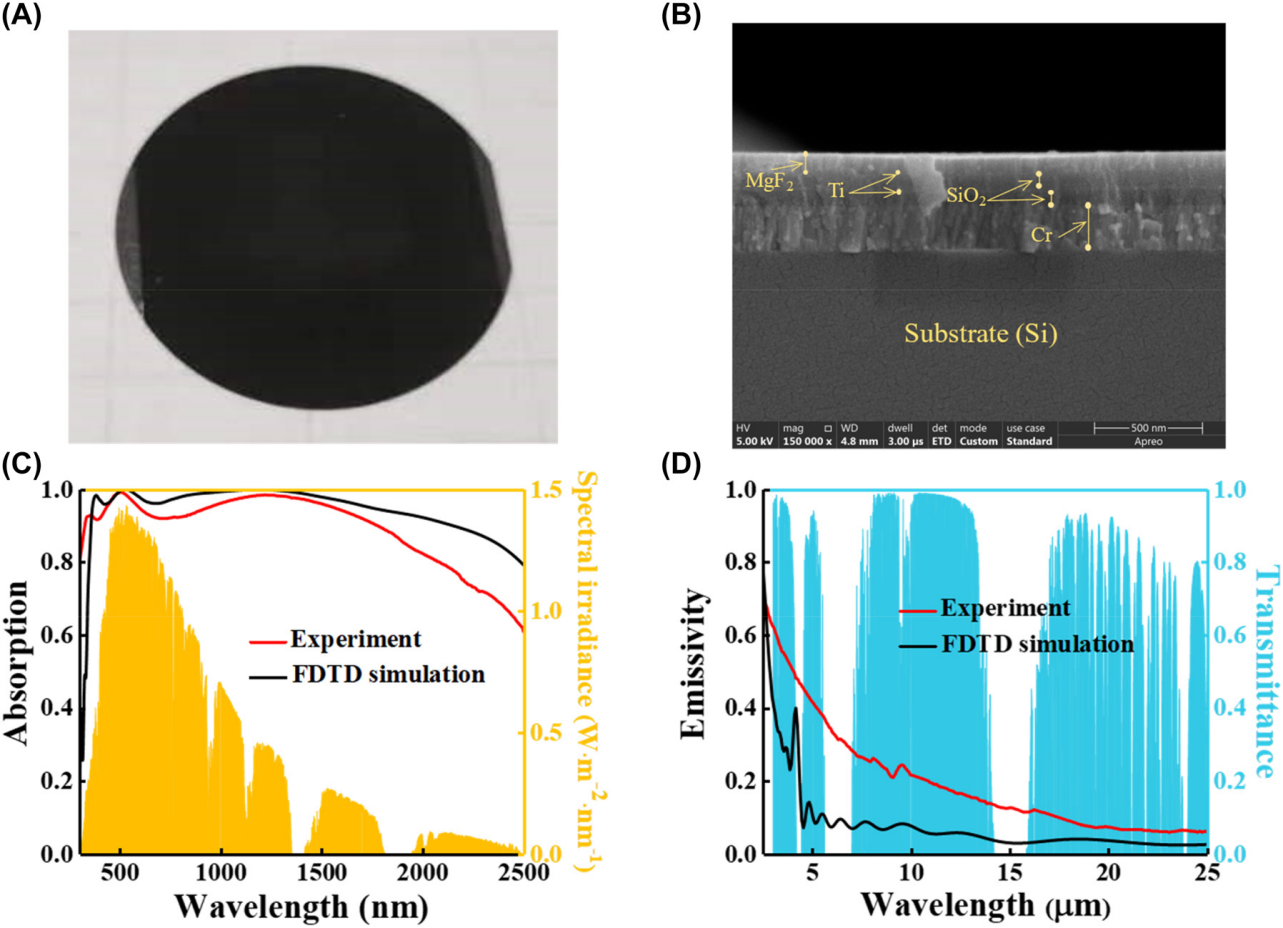
Morphological and spectral characterization of SSA. (A) A image of SSA made on a 2-inch silicon wafer; (B) the cross-section of the fabricated absorber as seen through SEM; (C) and (D) represent the absorption/emissivity of the fabricated SSA calculated by FDTD (black line) and experimentally measured (red line) in the solar spectrum and infrared region, respectively. The AM1.5 global tilted solar spectrum and atmospheric transparency window are provided as the background.

Based on the above design study, we fabricate the SSA sample and perform the characterization and optical measurement. We have successfully fabricated the designed six-layer absorber (MgF_2_/Ti/SiO_2_/Ti/SiO_2_/Cr) using a high vacuum electron beam evaporation thin film deposition system. Our wafer-scale SSA samples, as shown in Figure 3A, demonstrate this achievement. Furthermore, a cross-section of the fabricated absorber, depicted in Figure 3B through a scanning electron microscope (SEM), clearly shows each layer in sequence. These collective findings establish the successful fabrication of the SSA. The absorption/emissivity of the fabricated SSA calculated by FDTD and experimentally measured are shown in Figure 3C and D. With the best geometrical parameters and material combinations, the designed SSA has high absorption in the solar spectrum (calculate/measure = 0.98/0.94) and low average emissivity in the infrared region (calculate/measure = 0.08/0.19). The comparison between the measured absorption and the numerical simulation demonstrated good agreement, with minor variations potentially attributed to differences in thickness between the simulation and experiment, as well as the accumulated roughness during the fabrication process of the multilayer films leading to discontinuities in the ultrathin Ti layer (see more details in Figure S4, Supporting Information). These results provide further evidence of the success of the SSA design and fabrication. In addition, the designed absorber has an excellent incident-angle rudeness (see more details in Figure S5, Supporting Information).

To further demonstrate the superiority of our designed SSA, we compared its main spectral properties with those of current state-of-the-art solar absorbers, as shown in Table 1. Despite the experimental defect that resulted in a lower FOM than predicted, our fabricated SSA still exhibits the best spectral selectivity among all experimentally demonstrated solar absorbers reported in the literature. This represents a significant improvement over current state-of-the-art solar absorbers.

**Table 1:** Performance of the selective absorbers for solar spectral absorption.

Ref.	[31]	[14]	[43]	[44]	This work
Material	SiO_2_/W/Al_2_O_3_	Graphene/SiO_2_/Ag	TiN/SiN_x_	AlCrSiO_2_/TiN	MgF_2_/Ti/SiO_2_/Cr
Structure	Metamaterial	Metamaterial	Metamaterial	Layered film	Layered film
Absorption bandwidth (nm)	300–2500	300–2500	250–2250	300–2500	300–2500
Absorption (%)	93.2 (calculate)	85 (measure)	87 (measure)	∼92.3 (measure)	98.1 (calculate) 94.2 (measure)
Emission bandwidth (µm)	2.5–25	/	5–13	2.5–25	2.5–25
Emissivity (%)	5.8 (calculate)	/	29 (calculate)	∼16 (measure)	8.7 (calculate) 19.7 (measure)
Angular stability (Fom_1_ > 80 %)	∼75°	>60°	>60°	/	>75°
Origin of results	Simulation	Experiment simulation	Simulation	Experiment simulation	Experiment simulation
Design scheme	Iterative optimization	Brute-force simulation	Iterative optimization	Brute-force simulation	Deep learning

Note: ‘/’ denotes the absence of relevant research.

**Figure 4:**
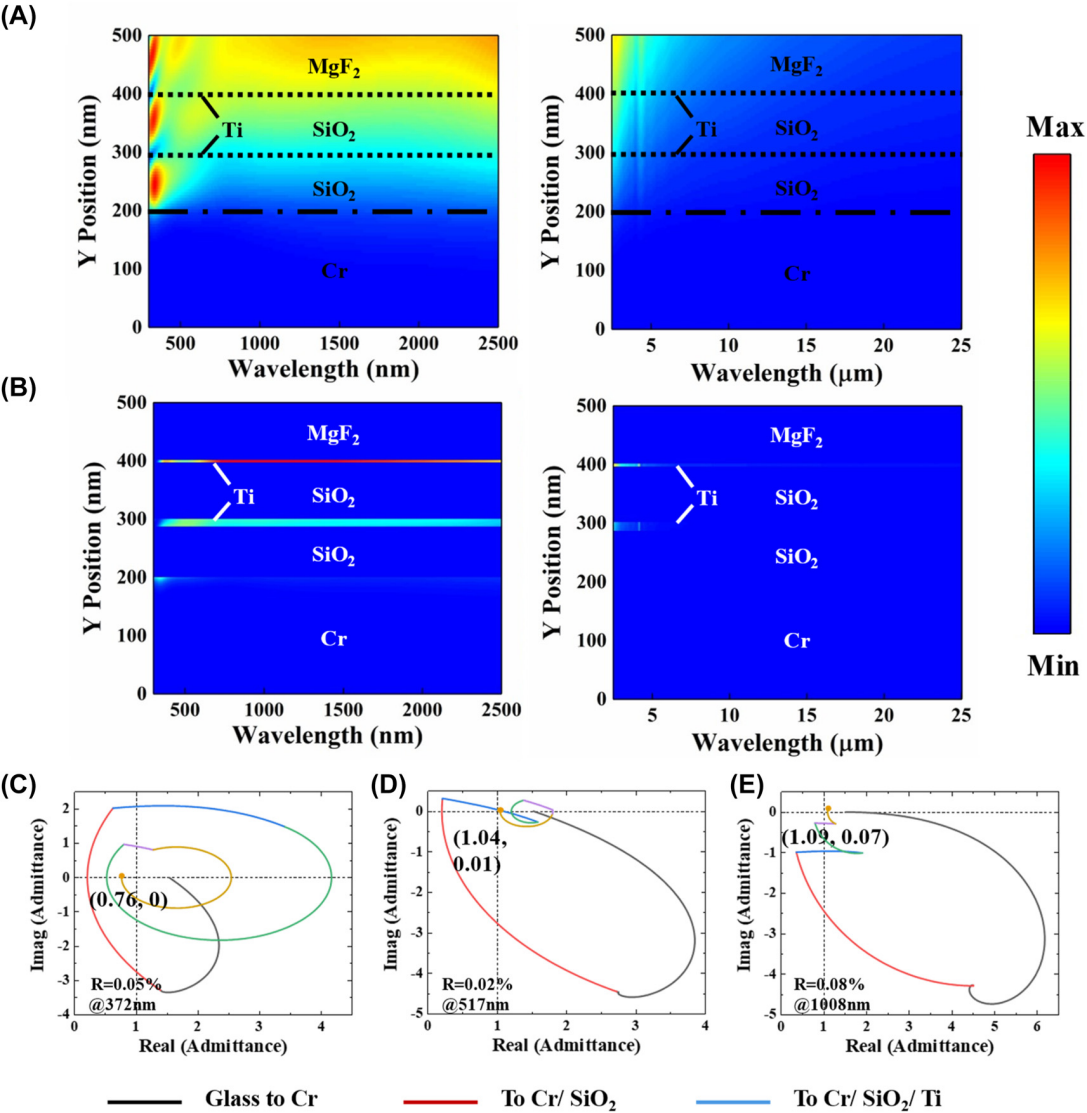
Simulated contour plots of (A) $\left|E\right|$ and (B) *P*_abs_ in the cross-section of the ISSA in the solar spectral to the infrared region; admittance loci diagrams of the ISSA at (C) 372 nm, (D) 517 nm, and (E) 1008 nm wavelengths.

The designed absorber exhibits a unique structure and material composition that contributes to its superior spectral properties. Specifically, the low refractive index MgF_2_ layer on the top acts as an anti-reflection layer, which minimizes the reflection of the incident light and maximizes the light that enters the absorber. The ultra-thin metal Ti [45], with its low-quality factor, serves as a translucent mirror that, in combination with the lossless dielectric SiO_2_ and the bottom Cr substrate, forms the MIMIM structure. This combination of layers and materials is responsible for the exceptional performance of the absorber. This structure forms two low-quality factor asymmetric Fabry–Perot resonators [46]. The FOM_1_ of different anti-reflection and bottom layer materials on the absorber performance is shown in Figure S6 (Supporting Information). In order to reveal the physical mechanism of the designed absorber’s ultra-broadband perfect absorption, we calculated the electric field intensity distribution ( $\left|E\right|$ ) and power absorption distribution (*P*_abs_) in the *X*–*Z* plane of the absorber calculated by FDTD method. Figure 4A and B shows the simulated contour plots of $\left|E\right|$ and *P*_abs_ as a function of wavelength and thickness of the designed absorber, respectively. As shown in Figure 4A, at short wavelengths, the light has a high penetration capacity and can enter the interior of the lower cavity and form standing waves. When the incident light wavelength increases, the light penetration ability into the MIM cavity becomes weaker and the intensity of the cavity mode is weakened [47]. The decrease in electric field intensity within the SiO_2_ spacer layer confirms the low-quality factor of the Fabry–Perot resonators [48]. The power absorbed by a nonmagnetic material can be described using the following expression: $P_{\text{abs}}=\nabla \vec{s}=\frac{1}{2}\omega \varepsilon^{\prime \prime }{\left|E\right|}^{2}$ , where *ɛ*″ is the imaginary portion of the permittivity, *ω* is the angular frequency, and $\left|E\right|$ is the total electric field distribution [49]. It is evident from Figure 4B that the top Ti layer contributes significantly to the absorption in the solar spectrum. The power absorbed by the middle Ti layer is lower than that absorbed by the top. The Cr substrate absorbs almost nothing except at short wavelengths, indicating that it is only useful for reflection (or non-transmission). Thus, the two Ti layers are primarily responsible for the solar spectrum’s high absorption.

Since the thicker Cr metal layer has no transmitted light and high absorption corresponds to low reflectivity, the mechanism of the designed absorber to achieve broadband absorption can also be investigated by optical admittance diagram at different wavelengths. The optical admittance is the inverse of the impedance, which is expressed by the equation $Y=\sqrt{\frac{\varepsilon }{\mu }}$ , where *ɛ* and *μ* are permeability and permittivity, respectively [50]. The optical admittance of the designed absorber starts at the point of the substrate (*n*_Cr_, 0). Variations in both material refractive index and film thickness cause changes in spiral trajectory and circular. When the admittance of the absorber matches exactly with the admittance of the air ( $R\left(\lambda \right)=0$ ), the trace of the admittance ends at the air point (1, 0). Therefore, for a layered photonic structure of the absorber, the distance between the termination admittance point and the air point determines the reflectivity of the absorber. Figure 4C and E show the optical admittance diagrams of the designed SSA at 372 nm, 517 nm, and 1008 nm (These wavelengths correspond to the absorption peaks), corresponding to the final admittance points of (0.76, 0), (1.04, 0.01), and (1.09, 0.07), corresponding to 0.05 %, 0.02 %, and 0.08 %, respectively. The top MgF_2_ layer can effectively reduce the final admittance of the absorber, thus producing broadband anti-reflection effects, which ultimately reduces the reflectance of the entire structure.

**Figure 5:**
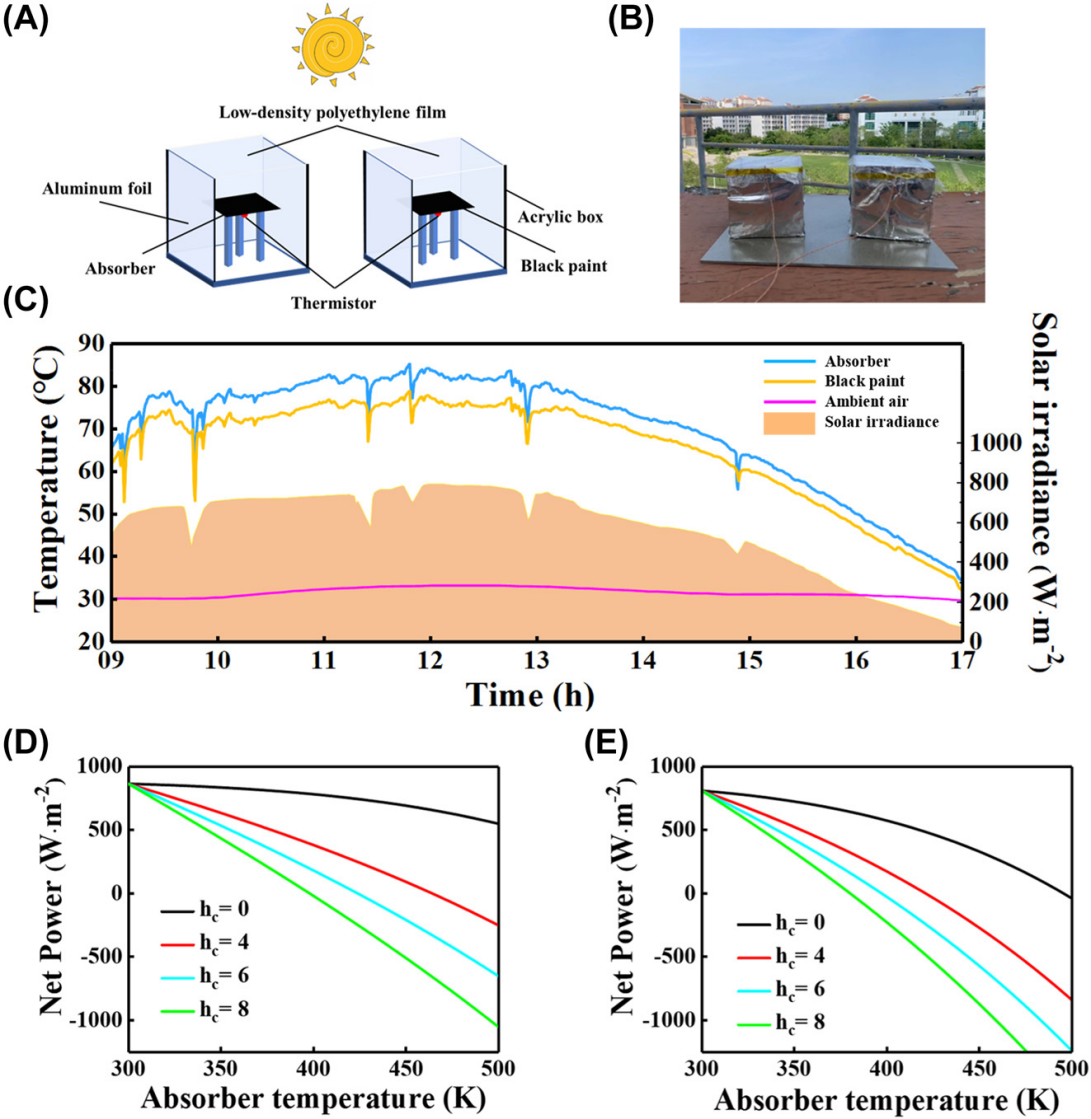
Schematic (A) and photograph (B) of the outdoor temperature measurement chamber; (C) the measured surface temperature of the two samples as a function of time when exposed on a sunny day in Xiamen, Fujian, China (09:00–17:00, September 28, 2022). The yellow area indicates the measured solar irradiance, corresponding to the right axis. The calculated net power as a function of temperature based on the absorption/emissivity spectra of the simulated (D) and measured (E) results with four different values of heat transfer coefficients.

We further perform a series of on-site experiments to evaluate the application capability of our SSA. The experiments were performed on the roof of the Keli Building of Jimei University, Xiamen, Fujian, China, on September 28, 2022. The illustration and photographs of the heating measurement system used in the experiment are shown in Figure 5A and B, respectively. As shown in Figure 5A, the SSA and the black paint (the spectral properties of black paint are shown in Figure S7, Supporting Information) used as a control group were placed in two separate containers. The container is made of an acrylic box (size: 10 × 10 × 10 cm) with an opening at the top, which is surrounded by Al foil and covered with low-density polyethylene (LDPE) film at the top. Acrylic box can be used as a thermal insulation layer, Al foil can reflect solar radiation, and LDPE film can ensure that as much solar radiation as possible into the interior of the box while reducing the heat convection between the interior of the box and the outside environment, the purpose of this is to reduce the impact of heat convection and heat conduction in the environment on the absorber temperature. The transmittance spectrum of the LDPE film is shown in Figure S8 (Supporting Information). The sample in the container is supported by three wooden needles to reduce heat transfer from the bottom of the box to the sample, and a K-type thermocouple is placed at the bottom of the sample to monitor the real-time temperature. A solar radiation sensor is placed at the same height as the sample to monitor the real-time solar irradiance. The ambient air temperature is extracted from the Xiamen gaoqi international airport station [51].

Figure 5C shows the daytime temperature measurements accompanied by solar irradiance during the period 9:00–17:00. With the increase of solar irradiance, the temperature of both samples gradually increased and the temperature gap between them widened. When the solar irradiance peaked around noon (over 800 W/m^2^), both the absorber and the black paint also reached their maximum temperatures of 85.16 °C and 78.78 °C, respectively. The temperature of the two samples gradually approaches the ambient temperature as the solar radiance declines. The primary factor causing the temperature difference between the ideal absorber and the black paint is their different optical characteristics. The ideal solar absorber achieves superior warming performance with higher solar absorption and lower infrared emissivity. It is worth noting that the thickness of the ideal absorber is less than 500 nm while the black paint is above 20 µm. As the thickness of the black paint is challenging to control for quantitative analysis, we evaluated the heating performance of the two samples under ideal conditions.

In accordance with the first law of thermodynamics, *P*_net_ can be calculated as follows [42]:

(6) $$P_{\text{net}}\left(T_{s}\right)=P_{\text{solar}}+P_{\text{atm}}\left(T_{a}\right)-P_{\text{rad}}\left(T_{s}\right)-P_{\text{con}}\left(T_{s}\right)$$

where

(7) $$P_{\text{rad}}\left(T_{s}\right)=\int \cos\theta d\omega \overset{\infty }{\underset{0}{\int }}I_{bb}\left(\lambda ,T_{s}\right)\varepsilon \left(\lambda ,\theta \right)\text{d}\lambda$$

denotes the radiation power density by the absorber at a temperature of *T*_*s*_;

(8) $$P_{\text{atm}}\left(T_{a}\right)=\int \cos\theta d\omega \overset{\infty }{\underset{0}{\int }}I_{bb}\left(\lambda ,T_{a}\right)\varepsilon \left(\lambda ,\theta \right)\varepsilon_{\text{atm}}\left(\lambda ,\theta \right)\text{d}\lambda$$

is the absorbed heat flux due to surrounding atmospheric thermal radiation at a temperature of *T*_*a*_;

(9) $$P_{\text{solar}}=\overset{\infty }{\underset{0}{\int }}I_{\text{AM}1.5}\left(\lambda \right)\varepsilon_{\text{solar}}\left(\lambda ,\theta \right)\text{d}\lambda$$

denotes the incident solar power absorbed by the absorber;

(10) $$P_{\text{con}}\left(T_{s}\right)=h_{c}\left(T_{s}-T_{a}\right)$$

denotes the power loss caused by conduction and convection between the absorber and the ambient air. Additionally, $I_{bb}\left(\lambda ,T_{s}\right)=2hc^{2}/\left[\lambda^{5}\exp\left(\frac{hc}{\lambda kT_{s}}\right)-1\right]$ is the spectral radiance of a blackbody at temperature *T*_*s*_. *ε*(λ, θ) is the directional emissivity and spectral of the absorber. $\varepsilon_{\text{atm}}\left(\lambda ,\theta \right)=1-t{\left(\lambda \right)}^{1/\cos\theta }$ is the emittance of the atmosphere at a zenith angle of θ, where *t*(*λ*) is the atmospheric transmittance in the zenith direction. The $I_{\text{AM}1.5}\left(\lambda \right)$ [42] denotes the direct normal irradiance of solar light at the wavelength *λ* and *h*_*c*_ is the convective heat-transfer coefficient.

With the aim to validate these experimental findings, we evaluated the net power as a function of temperature based on the absorption/emissivity spectra of the simulated (Figure 5D) and measured (Figure 5E) results with four different values of heat transfer coefficients. The solar irradiance and atmospheric transmittance shown in Figure S9A and B (Supporting Information) are used. The net power of the simulated results shows a higher net power than that of the measured results, which matches the averaged optical properties presented in Figure 3. At an ambient temperature of 300 K, the calculated net powers of the simulated and measured results were 861.81 and 807.55 W/m^2^, respectively. When the net power is equal to zero (*P*_net_ = 0), the absorber reaches the steady-state temperature. The steady-state temperatures at different heat transfer coefficients are shown in Table 2, and the steady-state temperature of the absorber gradually decreases as the heat transfer coefficient increases. The net power of black paint as a function of temperature calculated from its spectral properties is shown in Figure S10 (Supporting Information). Due to the larger emissivity of black paint in the infrared region, excessive heat leakage is generated, which greatly reduces its steady-state temperature. The steady-state temperature of black paint with different heat transfer coefficients is shown in Table S2 (Supporting Information).

**Table 2:** The calculated steady-state temperature of SSA under different heat transfer coefficients.

Steady-state	*h*_*c*_ = 0	*h*_*c*_ = 4	*h*_*c*_ = 6	*h*_*c*_ = 8
temperature (*P*_net_ = 0)				
Simulated result	>500 K	463 K	423 K	397 K
Measured result	495 K	420 K	397 K	380 K

**Figure 6:**
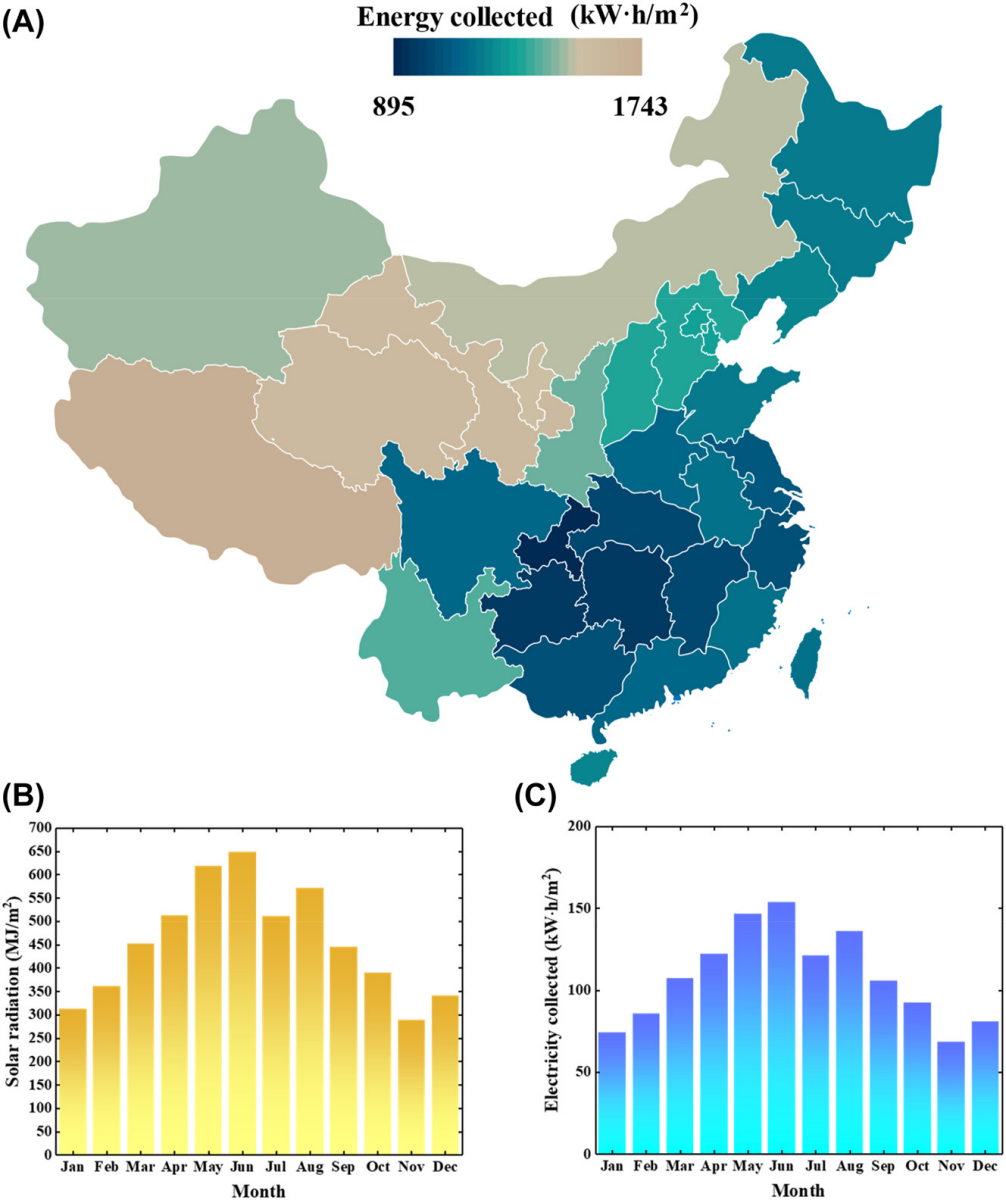
Energy harvesting potential of SSA. (A) Estimated energy collected if our designed SSA as a potential absorber material across the China; (B) the monthly variation of solar radiation in Xiamen; (C) electricity (energy) collected spectrum versus months.

We estimated the benefit of the SSA as a potential absorber material by calculating the energy it can collect, according to the assessment methods in Ref. [52] in Figure 6. The average annual energy collected over the surveyed Chinese provinces is 1461 kW h/m^2^. In high altitude provinces with hot and dry weather (e.g., Tibet), the SSA can potentially collected ∼1743 kW h/m^2^ per year (Figure 6A). The same calculation was also performed for the city of Xiamen. We refer to the monthly variation of solar radiation in Xiamen City [53] to objectively estimate the energy collected in Figure 6B, suggesting solar radiation in July is the highest and that in February is the lowest. We plot the estimated energy collected by the SSA every month in Figure 6C. Although this is a rudimentary analysis, it does indicate that more kilowatt hours can be collected in the summer months when there is more abundant solar radiation, and that the scale of the potential annual savings (1295 kW h/m^2^) is substantial.

## 3 Conclusions

In conclusion, we have successfully developed a high-performance SSA utilizing a DL- aided multi-objective DA algorithm optimization scheme. Our optimized SSA exhibits superior absorption and spectral selectivity (FOM_1_ = 0.94, FOM_2_ = 0.19) compared to other similar absorbers. Through experimental demonstration, we have validated the optical properties and heating performance of our SSA. Simulations based on the measured optical properties have shown the significant potential of our SSA in solar energy harvesting compared to black paint. As the fabrication of the optimized layered photonic structures in the SSA can be achieved using deposition techniques, it can potentially be scaled up for practical applications. Moreover, the DL-aided multi-objective DA algorithm optimization scheme employed in this study can be widely applicable for material design in various fields, including optical, thermal, and mechanical applications of meta-materials.

## 4 Experimental section

### 4.1 Numerical simulations

The numerical simulations were calculated with the FDTD Solutions v8.13, Lumerical software. The plane waves along the *z*-axis propagated to the solar absorber in the simulation. The boundary conditions along the *x*-axis and *y*-axis were periodic, and the boundary conditions along the *z*-axis were perfectly matched layers. In the simulation, the electromagnetic waves were introduced vertically incident on the SSA, and the normalized reflectance was collected with a frequency-domain field and power monitor placed behind the excitation source.

### 4.2 Data collection

A total of 30,000 samples were collected using a desktop, among which 27,000 were for the training samples, and 3000 were for the testing samples (Windows10 operation system, GeForce RTX 3070 GPU, AMD Ryzen 9 5900HX CPU @ 3.3 GHz and 16 GB of RAM). The geometry ranges can be found in Table S1 (Supporting Information). The model was constructed based on the open-source machine learning framework of TensorFlow. The version used was Python 3.9.

### 4.3 Training hyperparameters

The hyperparameters of the MLP structure are tuned by the Optuna framework. Optuna is a powerful hyperparameter optimization library that automates the process of finding optimal hyperparameter settings. In the Optuna framework, the range and type of hyperparameters to be adjusted are predefined. This included the number of hidden layers and the number of neurons per layer in the MLP architecture. We set the search space for the number of hidden layers to be from 1 to 8, and the search space for the number of neurons per layer to be (32, 64, 128, 256, 512, 1024, 2048). The performance of the MLP network using a given configuration of hyperparameters was evaluated by using the loss function on the test set as the objective function.

### 4.4 Sample fabrication

We fabricate the designed six-layer absorber (MgF_2_/Ti/SiO_2_/Ti/SiO_2_/Cr) with the physical vapor deposition method using a high vacuum electron beam evaporation thin film deposition system (DZS-500, Shenyang Scientific Instrument Co., Ltd., China). The evaporation materials, such as 99.99 % MgF_2_, Ti, SiO_2_, and Cr pellets, are purchased from ZhongNuo Advanced Material (Beijing) Technology Co., Ltd. Prior to deposition, the silicon substrate (size: 2 inches) is pre-processed in a clean room by ultrasonic cleaning using ethanol, acetone, isopropyl alcohol, and demineralized water, respectively, in sequence. After blowing dry with nitrogen, the silicon substrate adheres to the substrate holder. Throughout the deposition process, 2.0 × 10^−4^ Pa of pressure was kept in the vacuum chamber. To guarantee the uniformity of the films, the substrate holder was rotated at 15 rpm in a clockwise direction. For MgF_2_, SiO_2_, Ti, and Cr, the films’ deposition rates were kept under control at roughly 0.55 Å/s, 0.45 Å/s, 0.4 Å/s, and 0.5 Å/s, respectively. The INFICON STM-2XM thickness detector was used to measure the deposited films’ thickness in real-time.

### 4.5 Material characterizations

The cross-section morphologies and EDS were characterized by SEM (Sigma 300, Zeiss, Germany).

### 4.6 Optical characterization

The UV–visible–NIR (0.3–2.5 µm) reflectance (R) spectra of the samples were measured using a spectrometer (Shimadzu UV-3600i Plus) equipped with a 150 mm integrating sphere. The infrared (2.5–25 µm) emissivity of the sample was measured by a Fourier infrared spectrometer (Nicolet iS50, Thermo Scientific, USA) with a gold integrating sphere. The absorptance (A) spectra were directly derived from 1 – R – T. According to Kirchhoff’s law at thermal equilibrium, the spectral emissivity (*ε*(*λ*)) and absorptance are equal at any specified temperature and wavelength (*ε*(*λ*) = *α*(*λ*)) [54, 55].

### 4.7 Thermal measurement

The outdoor solar radiation intensity *P*_solar_ was measured by a photoelectric solar radiation sensor (RS-RA-N01-AL, Shandong Renke Control Technology Ltd. Co., China). The temperature of the sample was measured by a contact thermocouple thermometer (UT325, Uni-Trend Technology Ltd. Co., China).

## Supplementary Materials

See Supplementary Materials for supporting content.

## Supplementary Material

Supplementary Material Details
