# Supplementary material for: Deep learning empowering design for selective solar absorber
Source: Nanophotonics. 2023 Aug 11;12(18):3589–601. doi: 10.1515/nanoph-2023-0291 (PMC11502052; doi:10.1515/nanoph-2023-0291)
Supplement: Supplementary file 1 — Supplementary Material Details [file j_nanoph-2023-0291_suppl_001.pdf]

Supporting Information

Deep Learning Empowering Design for Selective Solar Absorber

Wenzhuang Ma[1], Wei Chen[2], Degui Li[3], Yue Liu[3], Juhang Yin[1], Chunzhi Tu[1], Yunlong Xia[1], Gefei Shen[1], Peiheng Zhou[1], Longjiang Deng[1], Li Zhang[1,*]

[1]*National Engineering Research Center of Electromagnetic Radiation Control Materials, Key Laboratory of Multi-spectral Absorbing Materials and Structures of Ministry of Education, University of Electronic Science and Technology of China, Chengdu, 611731, China*

[2]*Institute of Electromagnetics and Acoustics and Key Laboratory of Electromagnetic Wave Science and Detection Technology Xiamen University Xiamen, Fujian 361005. China*

[3]*School of Ocean Information Engineering, Jimei University, Xiamen 361021, China*

*Corresponding authors:
E-mail address: lzhang129@uestc.edu.cn (L. Zhang)

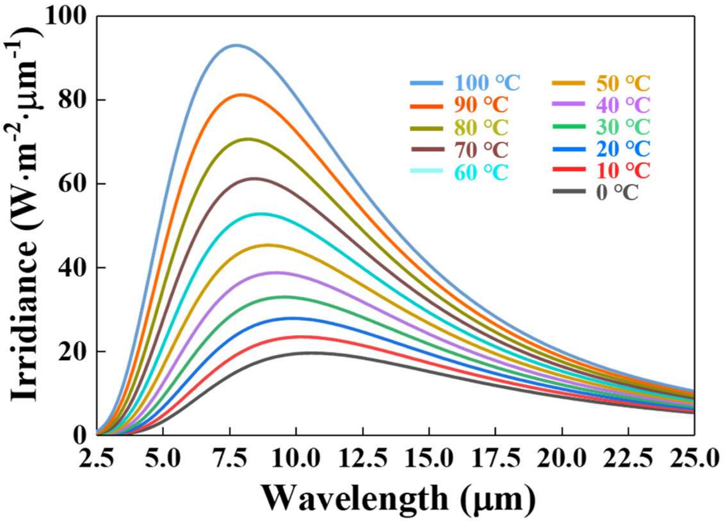

Fig. S1. Blackbody radiation spectra at T = 0-100 ℃.

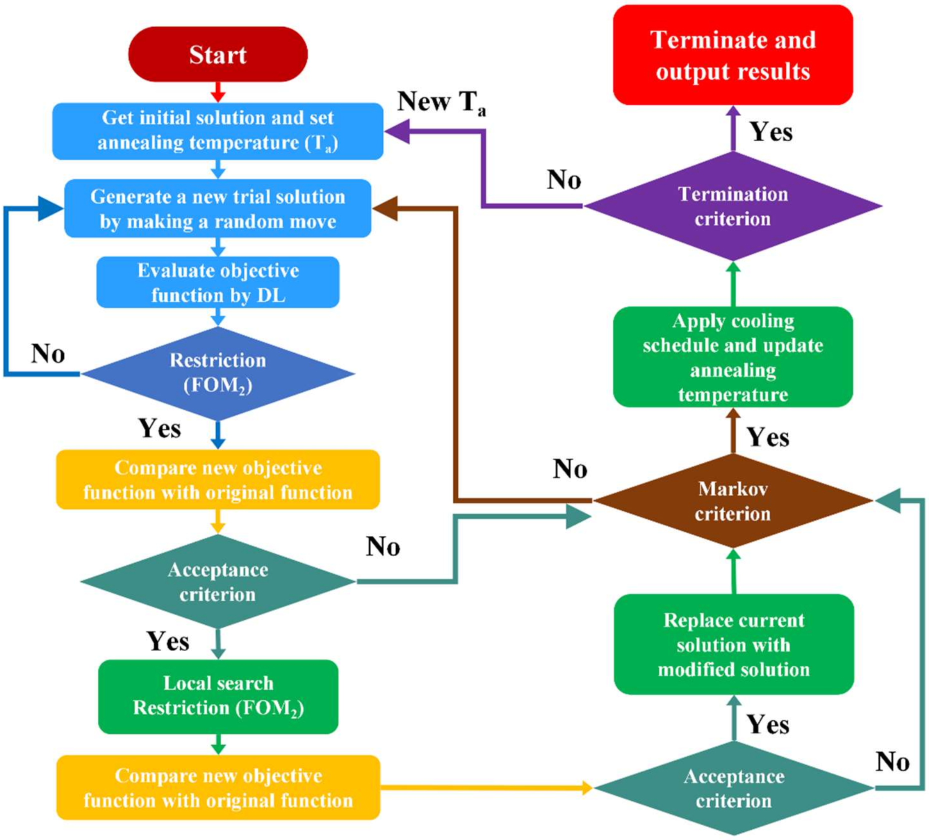

Figure. S2. Flow diagram of optimizing the SSA using multi-objective double annealing algorithms.

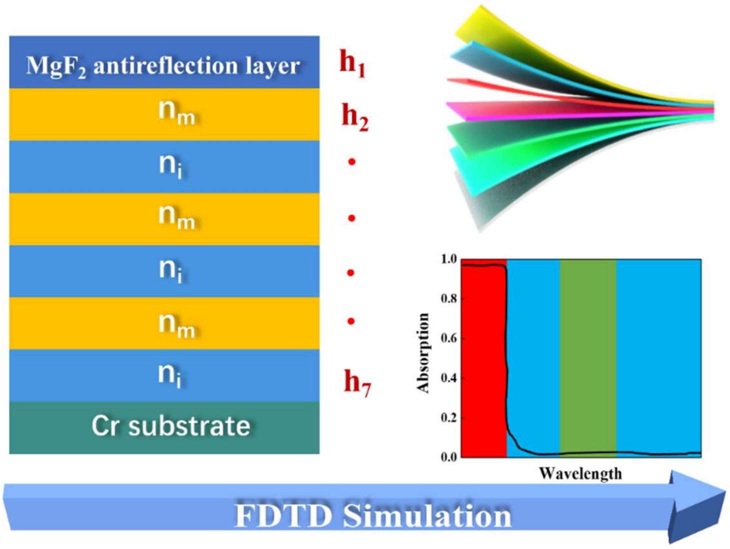

Figure. S3. The schematic of constructing a dataset.

Any DL network needs a dataset that contains relevant examples during the training phase, which is essential. The dataset was created using the FDTD method, which accurately calculates the absorption spectrum of layered photonic structures. The spectral transmittance ($T(\lambda)$) and reflectance ($R(\lambda)$) of the proposed absorber are monitored in the simulation. We calculate the absorption ($\alpha(\lambda)$) as follows: $\alpha(\lambda) = 1 - T(\lambda) - R(\lambda)$. Table. 1 lists the structure, layers, potential materials, and range of thicknesses that were used to create the dataset. As shown in Fig. 1, this paper selects the metal-insulator-metal (MIM) layered film photonic structure to design the ISSA, taking into account the need for large-area preparation in real-world industrial applications. Similar structures have been extensively demonstrated to achieve ideal solar absorption through Fabry-Perot resonance and anti-reflection effects [1, 2]. The Magnesium fluoride (MgF_2) anti-reflection layer covers the top of the structure, and the Chromium (Cr) layer at the bottom has a default thickness of 200 nm, which is greater than the skin depth. The thickness ranges of the insulator (n_i) and metal (n_m) layers are

set to 0-300 nm and 0-30 nm, respectively. Among many possible materials in nature, we selected Aluminum oxide (Al_2O_3), MgF_2, and Silicon dioxide (SiO_2) [3-5] as dielectric materials, while the Iron (Fe), Titanium (Ti), Tungsten (W), and Cr [6-8] were chosen as metallic materials because of their excellent optical properties and durability. The chosen twelve material combinations were then indexed for material optimization. Create 30,000 random thickness combinations with a thickness accuracy of 1 nm for each combination of materials. A total of 360,000 simulations were performed to prepare the dataset in lieu of more than 10^{15} possible combinations that could exist. The solar spectrum (0.3-2.5 μm) was discretized into 2201 equally spaced data points and the infrared region (2.5-25 μm) was discretized into 300 equally spaced data points.

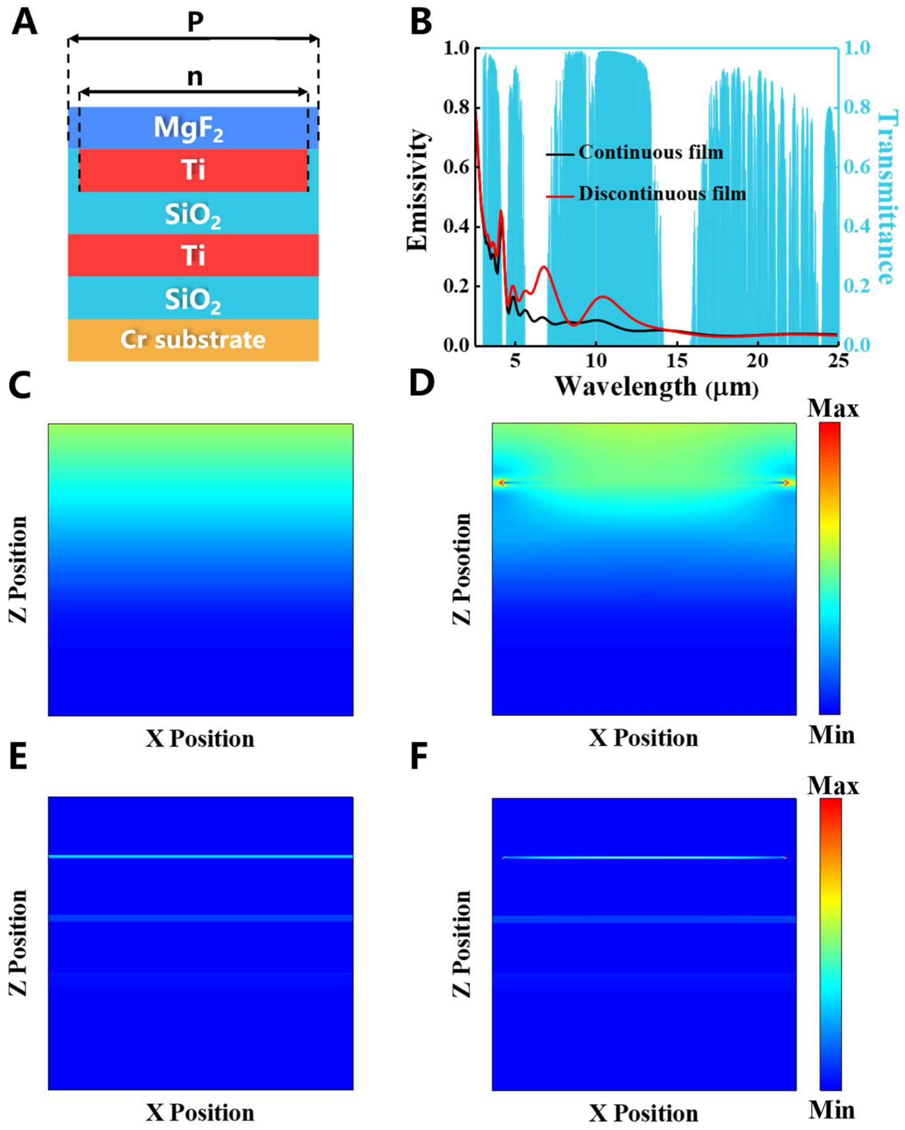

Figure. S4. (a) Cross-sectional schematic of the simulated structure model with a discontinuous Ti layer, where the FDTD calculation period p = 300 nm and the discontinuous Ti layer calculation period n = 280 nm; (b) The infrared emissivity of the continuous Ti layer and the discontinuous Ti layer calculated through FDTD simulation; The $|E|$ and P_{abs} of the continuous Ti layer (c and e) and the discontinuous Ti layer (d and f).

Due to the accumulated surface roughness of the multilayer film, the top ultra-thin Ti layer may not be continuous. Therefore, we further examined the potential influence of the top ultra-thin Ti layer's discontinuity on the infrared spectral response. To achieve this, we conducted FDTD simulations to assess the effect of the discontinuous Ti layer on the infrared emissivity, and the established model is shown in Fig. S4A. The results

indicate that the infrared emissivity significantly increases in the case of a discontinuous Ti layer compared to a continuous one. To further investigate the physical mechanism behind the increased infrared emissivity caused by the discontinuous Ti layer, we computed the electric field intensity distribution ($|E|$) and power absorption distribution (P_{abs}) (normalized electric field intensity and P_{abs}) for both continuous and discontinuous Ti layers at the 6.8 μm absorption peak. As shown in Fig. S4C and D, for the continuous Ti layer, the $|E|$ intensity decreases layer by layer after the incident electromagnetic wave; however, for the discontinuous Ti layer, localized effects are observed near the gaps, indicating the possible excitation of localized surface plasmon resonance (LSPR), which enhances the absorption of electromagnetic waves. The results from Fig. S4E and F also support this observation, as the discontinuous Ti layer contributes more to the absorption intensity compared to the continuous Ti layer.

In summary, the discrepancies between the infrared spectra and the measured spectra may arise from two main factors. Firstly, the presence of a non-continuous Ti layer on the top surface could trigger localized surface plasmon resonance (LSPR) phenomena, leading to enhanced infrared absorption. Secondly, the thickness error and material refractive index error during the preparation process also influence the spectral characteristics.

Table. S1. Details of multilayer structures used to create the dataset

Layer	Material	Thickness (nm)
n_{top}	MgF_2	0-300
n_m	Fe, Ti, W, Cr	0-30
n_i	Al_2O_3, MgF_2, SiO_2	0-300
n_{bottom}	Cr	200

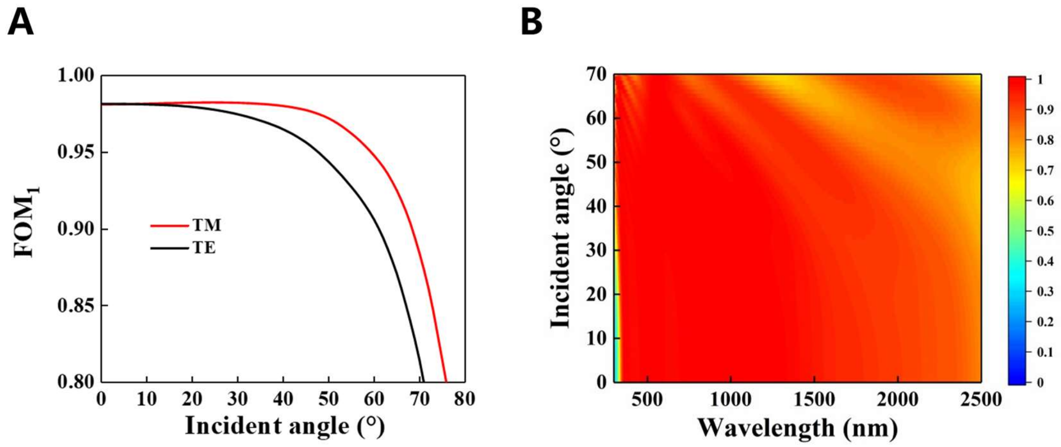

Figure. S5. (a) FOM_1 curve of the solar absorber with different incident angles both in TM and TE modes; (b) The simulated absorption spectra of the SSA in TM mode versus the wavelength of the incident light.

Fig. S5A shows the calculated FOM_1 for both TM and TE modes. The results show that the SSA maintains high efficiency ($FOM_1 > 0.9$) of absorption from 0° to 60° in both TE and TM modes. Even when the incidence angle is greater than 70°, the FOM_1 of the TE and TM modes still exceeds 0.8. In order to clearly understand the influence of incident angle on the absorption efficiency of our proposed absorber, we plot the absorption evolution spectra with the incident angles from 0° to 70° at the TM model, as shown in Fig. S5B. The results show that with the increase of the incident angle, the absorption at the long wavelength appears to decrease slightly, and the absorption at the short wavelength essentially stays the same. These features demonstrate that the designed absorber has excellent incident-angle rudeness.

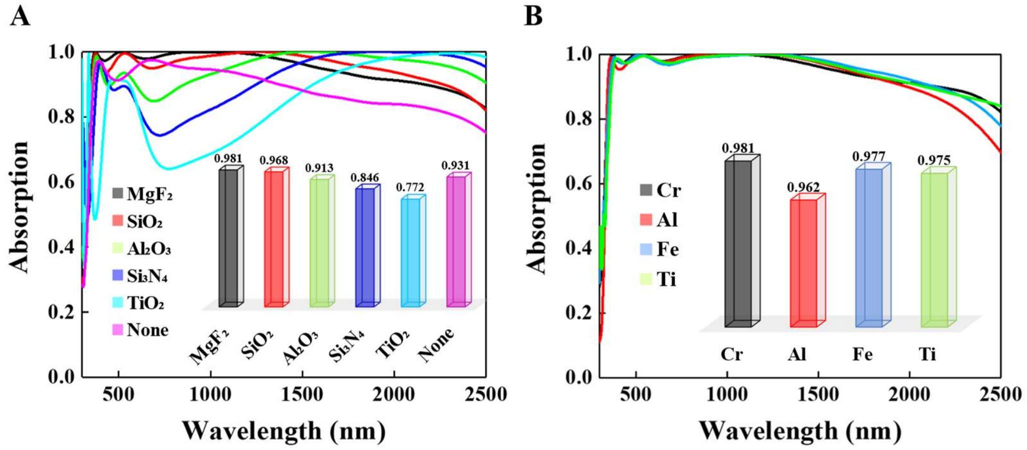

Figure. S6. Effects of variations in used materials on the FOM_1 of the designed SSA. (a) using MgF_2, SiO_2, Al_2O_3, Si_3N_4, and TiO_2 as anti-reflection layer and (b) using Cr, Al, Fe, and Ti as bottom metals.

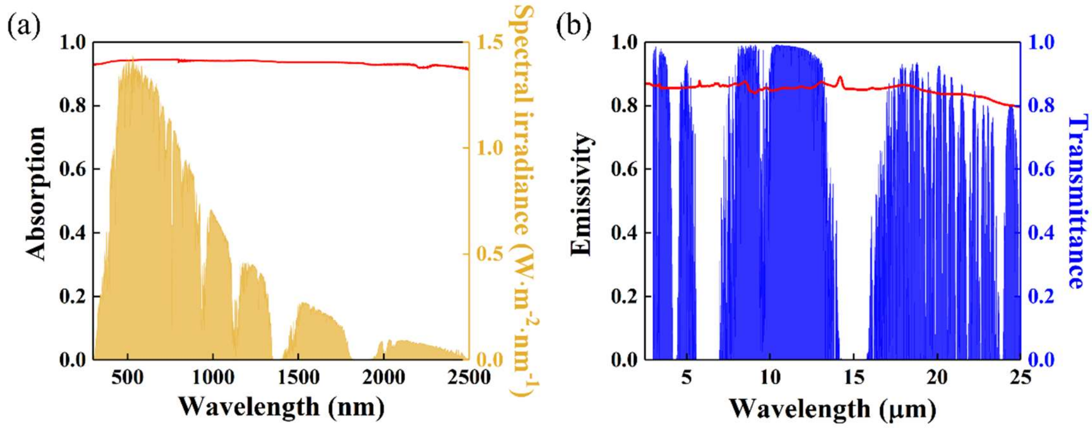

Figure. S7. Measured absorption of the commercial black paint in the solar spectrum (250-2500 nm) and the AM1.5 spectral irradiance spectrum are provided as the background. (b) The measured emissivity of the commercial black paint in the infrared region (2.5-25 μm) and the atmospheric transmittance are provided as background.

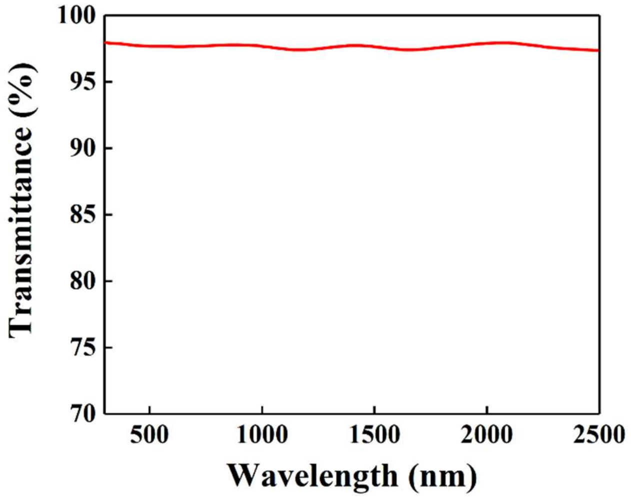

Figure. S8. The transmittance spectrum of the LDPE film

Fig. S8 shows the transmittance of LDPE film which was used in external heating temperature measurement. average transmittance values of LDPE film are 0.976 in the solar spectrum (0.3-2.5 μm).

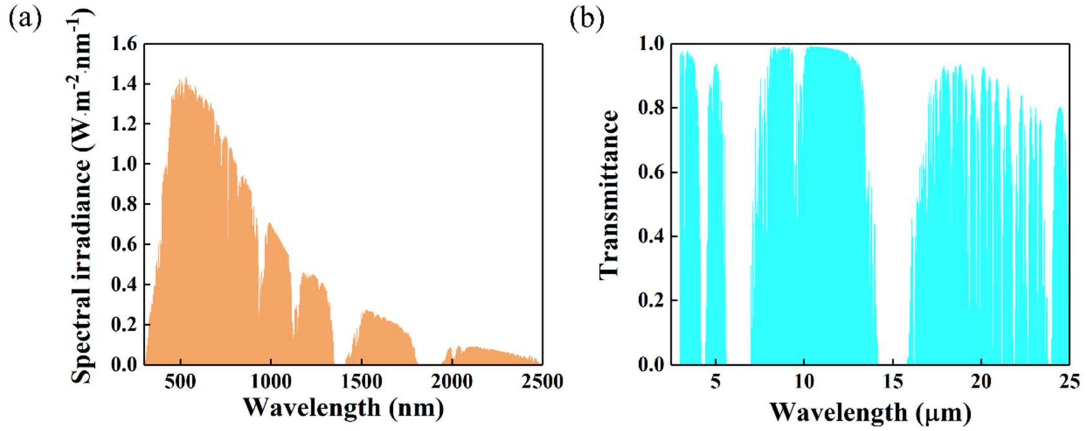

Figure. S9. (a) Solar irradiance (AM1.5 Direct) and (b) atmospheric transmittances versus wavelength from 2.5 to 25 μm (AM1.5 atm).

Figure S9A presents an irradiance graph which is AM1.5 Direct used for heating performance calculation. Net solar power density is 892 Wm^{-2}. Figure S9B shows the atmospheric transmittance used in the calculation process. The atmospheric transmittance values of AM1.5 atm were obtained from the Gemini Observatory website [9].

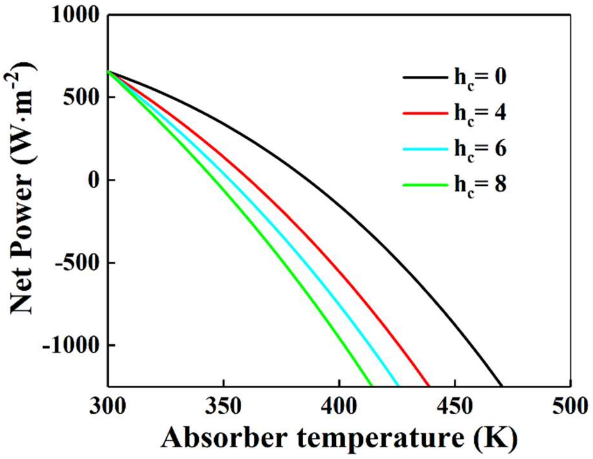

Figure. S10. The net power of black paint as a function of temperature calculated from its spectral properties.

Table. S2. The steady-state temperature of black paint with different heat transfer coefficients.

Steady-state temperature ($P_{net} = 0$)	$h_c = 0$	$h_c = 4$	$h_c = 6$	$h_c = 8$
Simulated result	386 K	361 K	352 K	346 K

References

[1] H. Cai, M. Wang, Z. Wu, X. Wang, J. Liu, Design of multilayer planar film structures for near perfect absorption in the visible to near-infrared. Opt. Express. 30 (20) (2022) 35219-35231

[2] W. Chen, Y. Gao, Y. Y. Li, Y. M. Yan, J.-Y. Ou, W. Z. Ma, J. F. Zhu. Broadband Solar Metamaterial Absorbers Empowered by Transformer-Based Deep Learning. Advanced Science, 2023, 2206718.

[3] H.S. Nalwa, Silicon-based material and devices, two-volume set: materials and processing, properties and devices., Academic Press, 2001.

[4] H.S. Nalwa, Handbook of thin film materials., Academic Press, 2002.

[5] E.D. Palik, Handbook of optical constants of solids academic press. Inc., New York (1985).

[6] A.D. Rakić, A.B. Djurišić, J.M. Elazar, M.L. Majewski, Optical properties of metallic films for vertical-cavity optoelectronic devices. Appl. Optics. 37 (22) (1998) 5271-5283.

[7] M.R. Querry, Optical constants, contractor report. US Army Chemical Research, Development and Engineering Center (CRDC), Aberdeen Proving Ground, MD. 418 ((1985).

[8] M.A. Ordal, R.J. Bell, R.W. Alexander, L.A. Newquist, M.R. Querry, Optical properties of al, fe, ti, ta, w, and mo at submillimeter wavelengths. Appl. Optics. 27 (6) (1988) 1203-1209.

[9] IR transmission spectra, Gemini Observatory, http://www.gemini.edu/sciops/telescopes-and sites/observing-condition-constraints/irtransmission-spectra.
